# Cassava geminivirus agroclones for virus-induced gene silencing in cassava leaves and roots

**DOI:** 10.1186/s13007-018-0340-5

**Published:** 2018-08-27

**Authors:** Ezequiel Matias Lentz, Joel-Elias Kuon, Adrian Alder, Nathalie Mangel, Ima M. Zainuddin, Emily Jane McCallum, Ravi Bodampalli Anjanappa, Wilhelm Gruissem, Hervé Vanderschuren

**Affiliations:** 1 Department of Biology, Plant Biotechnology, ETH Zurich-LFW, E56.1, Universitaetstrasse 2, 8092 Zurich, Switzerland; 2 Plant Genetics Lab, TERRA Research and Teaching Centre, Gembloux Agro BioTech, University of Liège, Gembloux, Belgium

**Keywords:** Cassava, VIGS, ACMV, Geminivirus, *Agrobacterium tumefaciens*, Gene silencing, Agroinoculation

## Abstract

**Aim:**

We report the construction of a Virus-Induced Gene Silencing (VIGS) vector and an agroinoculation protocol for gene silencing in cassava (*Manihot esculenta* Crantz) leaves and roots. The African cassava mosaic virus isolate from Nigeria (ACMV-[NOg]), which was initially cloned in a binary vector for agroinoculation assays, was modified for application as VIGS vector. The functionality of the VIGS vector was validated in *Nicotiana benthamiana* and subsequently applied in wild-type and transgenic cassava plants expressing the *uidA* gene under the control of the CaMV 35S promoter in order to facilitate the visualization of gene silencing in root tissues. VIGS vectors were targeted to the Mg2+-chelatase gene in wild type plants and both the coding and promoter sequences of the 35S::*uidA* transgene in transgenic plants to induce silencing. We established an efficient agro-inoculation method with the hyper-virulent *Agrobacterium tumefaciens* strain AGL1, which allows high virus infection rates. The method can be used as a low-cost and rapid high-throughput evaluation of gene function in cassava leaves, fibrous roots and storage roots.

**Background:**

VIGS is a powerful tool to trigger transient sequence-specific gene silencing *in planta*. Gene silencing in different organs of cassava plants, including leaves, fibrous and storage roots, is useful for the analysis of gene function.

**Results:**

We developed an *African cassava mosaic virus*—based VIGS vector as well as a rapid and efficient agro-inoculation protocol to inoculate cassava plants. The VIGS vector was validated by targeting endogenous genes from *Nicotiana benthamiana* and cassava as well as the *uidA* marker gene in transgenic cassava for visualization of gene silencing in cassava leaves and roots.

**Conclusions:**

The *African cassava mosaic virus*—based VIGS vector allows efficient and cost-effective inoculation of cassava for high-throughput analysis of gene function in cassava leaves and roots.

**Electronic supplementary material:**

The online version of this article (10.1186/s13007-018-0340-5) contains supplementary material, which is available to authorized users.

## Background

Validation of gene function in plants can be challenging in non-model species for which T-DNA insertion lines are not available and/or genetic transformation is time-consuming and inefficient. Therefore, reverse genetics tools such as virus-induced gene silencing (VIGS) are particularly useful when facing these limitations [1]. Initially, the VIGS technique was established to silence leaf-expressed genes [2, 3]. VIGS vectors were subsequently shown to effectively silence genes expressed in *Nicotiana benthamiana*, Arabidopsis and tomato roots [4] as well as potato tubers [5].

Cassava (*Manihot esculenta* Crantz) is one of the most important root crops worldwide, producing starchy roots used as staple food by more than 800 million people [6]. Over the last decade, the use of cassava for industrial applications has also increased [7, 8]. Although reliable cassava genetic transformation protocols are now available, transformation of cassava remains a lengthy process and several cassava varieties are recalcitrant to genetic transformation [9–12]. Therefore, efficient reverse genetic tools appear as particularly suitable to investigate the function of cassava genes for example those identified in large-scale omics studies [13–15]. Those studies are now facilitated by the release of cassava reference genome sequences [16–18].

Cassava mosaic geminiviruses are bipartite DNA begomoviruses causing the cassava mosaic disease (CMD) [19] and infecting a wide range of cassava varieties grown in Asia, South America and Africa [20–23]. We previously established *African cassava mosaic virus* isolate ACMV-[NOg] DNA-A and DNA-B clones to agro-inoculate cassava plants and screen varieties for CMD resistance [24]. Biolistic delivery of a modified Cameroon isolate ACMV-[CM] can trigger VIGS in cassava leaves [25]. Recently, a VIGS vector based on the *East African cassava mosaic virus* isolate EACMV-[K201] was developed to screen for virus resistance by biolistic delivery [26]. The high cost of commercial gene guns makes VIGS vectors relying on biolistic delivery methods not affordable to all labs [27]. Other methods for the inoculation of geminiviruses to plants include DNA abrasion [28] and Agrobacterium [24, 29, 30]. DNA abrasion is not suitable for all type of host plants [27, 28] and reports of successful geminivirus inoculation in cassava by abrasion have so far been scarce. On the contrary, efficient agro-inoculation with cassava geminiviruses agroclones has been previously established in cassava [24, 31]. Therefore, development of agroclones for VIGS in cassava appears to be a suitable strategy to enable the cost-effective use of this reverse genetics tool in cassava.

Because geminiviruses are detectable in the roots of tomato, Arabidopsis and cotton plants [32–36], we also hypothesized that cassava geminiviruses could sustain VIGS in cassava roots, further expanding the use of VIGS as a reverse genetic tool.

Here we present the development of a VIGS agroclone and the establishment of a robust and efficient inoculation protocol that leads to VIGS in cassava leaves as well as in fibrous and storage roots.

## Results

### VIGS vector construction, functionality check in *N. benthamiana* and cassava agro-inoculation

**Fig. 1:**
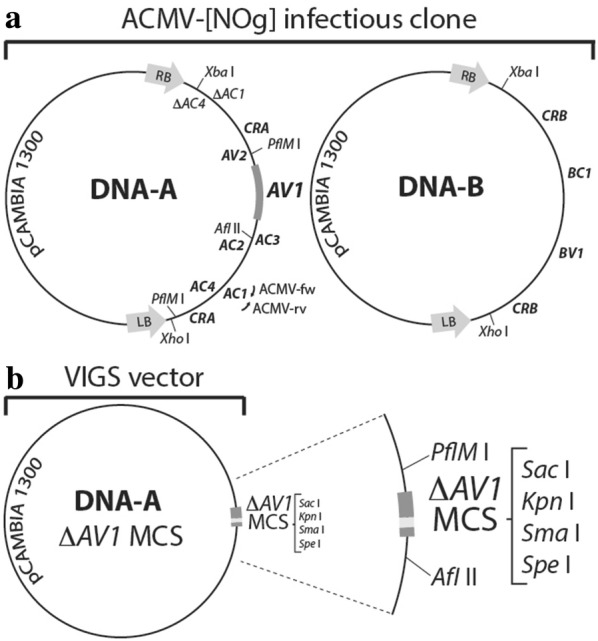
Construction of a VIGS vector based on the African Cassava Mosaic Virus (ACMV) infectious clone ACMV-[NOg]. **a** The genome of this bipartite geminivirus was previously cloned into two pCambia 1300 plasmids containing either the DNA-A or DNA-B ACMV-[Nog] sub-genomes *CRA*: Common Region of DNA-A; *AV1*: coat protein gene; *AV2*: Protein V2 gene; *AC3*: Replication enhancer protein gene; *AC2*: Transcriptional activator protein gene; *AC1*: Replication associated protein gene; *AC4*: RNA silencing suppressor gene [24]. **b** The cassava VIGS vector was constructed by modifying the DNA-A sub-genome by replacing part of the *AV1* gene with a 30-bp multiple cloning site (MCS) for insertion of gene targeting DNA fragments

**Fig. 2:**
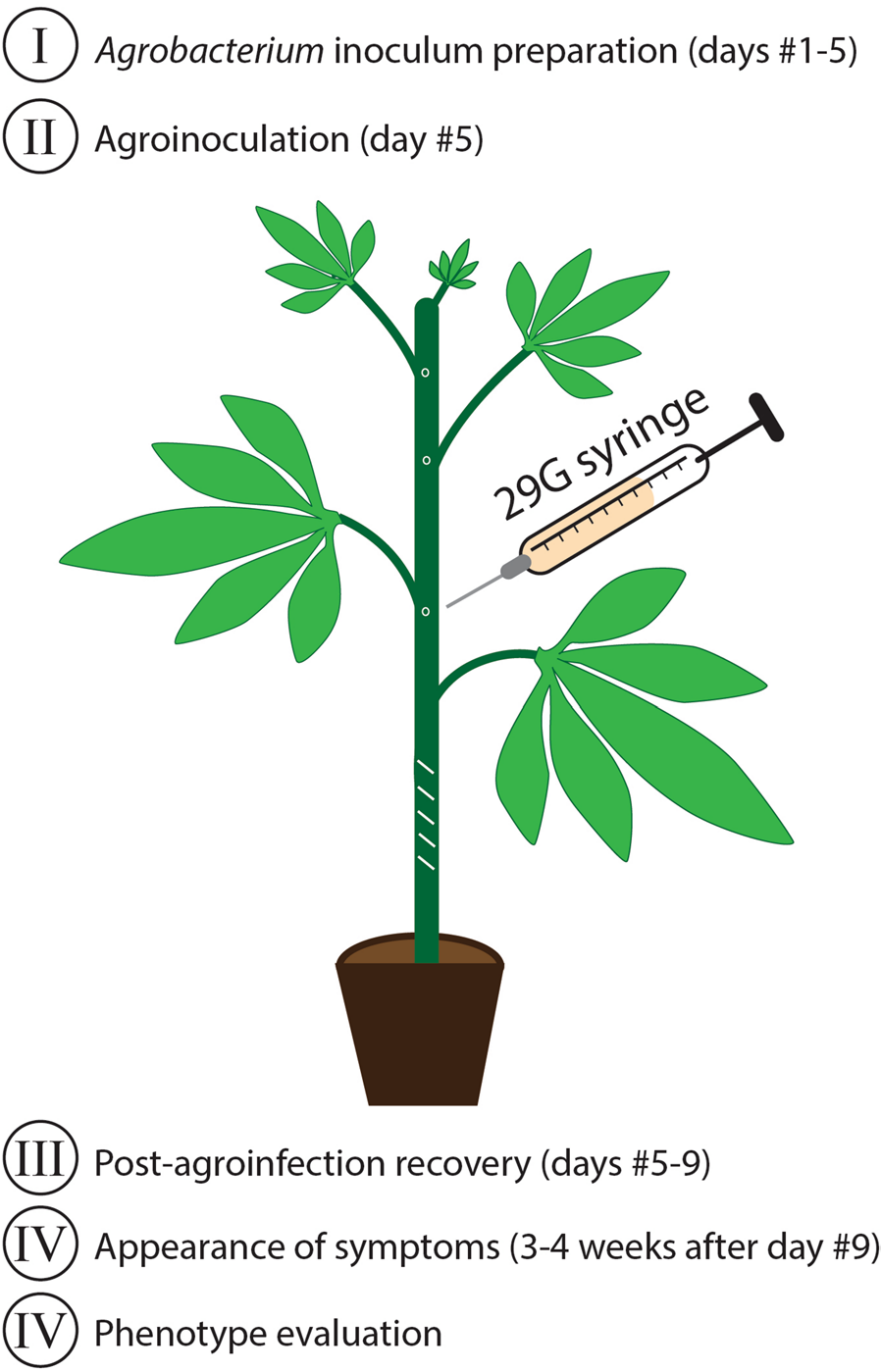
Overview of the VIGS assay in cassava. Six week-old cassava plants were injected with a 29G syringe filled with a suspension of mixed Agrobacteria containing either the DNA-A (VIGS vector) or DNA-B plasmids in at least three sites close to axillary meristems. Additional superficial cuts in the lower part of the stem were made using the same syringe to induce the T-DNA release from the Agrobacteria

We constructed a VIGS vector based on the DNA-A ACMV-[NOg] agroclone [24] by partial replacement of the coat protein sequence with a target sequence [3, 25, 36]. A 459 bp fragment of the ACMV *AV1* gene coding for the capsid protein was replaced by a 30 bp multiple cloning site (VIGS-MCS; Fig. 1 and Additional file 1: Fig. S1). The infectious agroclones assembled as tandem repeats in a binary vector were previously used for the evaluation of ACMV resistance in transgenic cassava as well as in wild-type cassava varieties [20, 24]). The infectivity of the VIGS-MCS agroclone along with the VIGS-*Chl1* construct, targeting the Mg^2+^-chelatase gene, were first confirmed in *Nicotiana benthamiana* using the *Agrobacterium tumefaciens* strain LBA4404. These DNA-A-based agroclones co-inoculated with the agroclone DNA-B resulted in 100% infection rates in *Nicotiana benthamiana*. We concluded that the infectivity of VIGS-MCS and VIGS-*Chl1* agroclones did not differ from the non-modified ACMV DNA-A agroclone in *Nicotiana benthamiana* (Additional file 1: Fig. S2 and Table S1 A, C and D). Inoculation of the agroclones was subsequently tested on wild-type cultivar 60,444 cassava plants propagated via stem cuttings and grown in the greenhouse. We adapted a previously established method [24] to agro-inoculate cassava (Fig. 2). Because agro-inoculation with the VIGS-MCS and VIGS-*Chl1* agroclones using the LBA4404 strain did not result in symptomatic cassava (Additional file 1: Table S1 C and D), the agroclones were transferred to the hyper-virulent strain AGL1. Performing the agro-inoculation with the AGL1 strain, infection rates of 27% and 92% were recorded for the VIGS-MCS agroclone and the VIGS-*Chl1* agroclone respectively (Additional file 1: Table S1 C and D).

### Visualization of VIGS in cassava leaves and roots

**Fig. 3:**
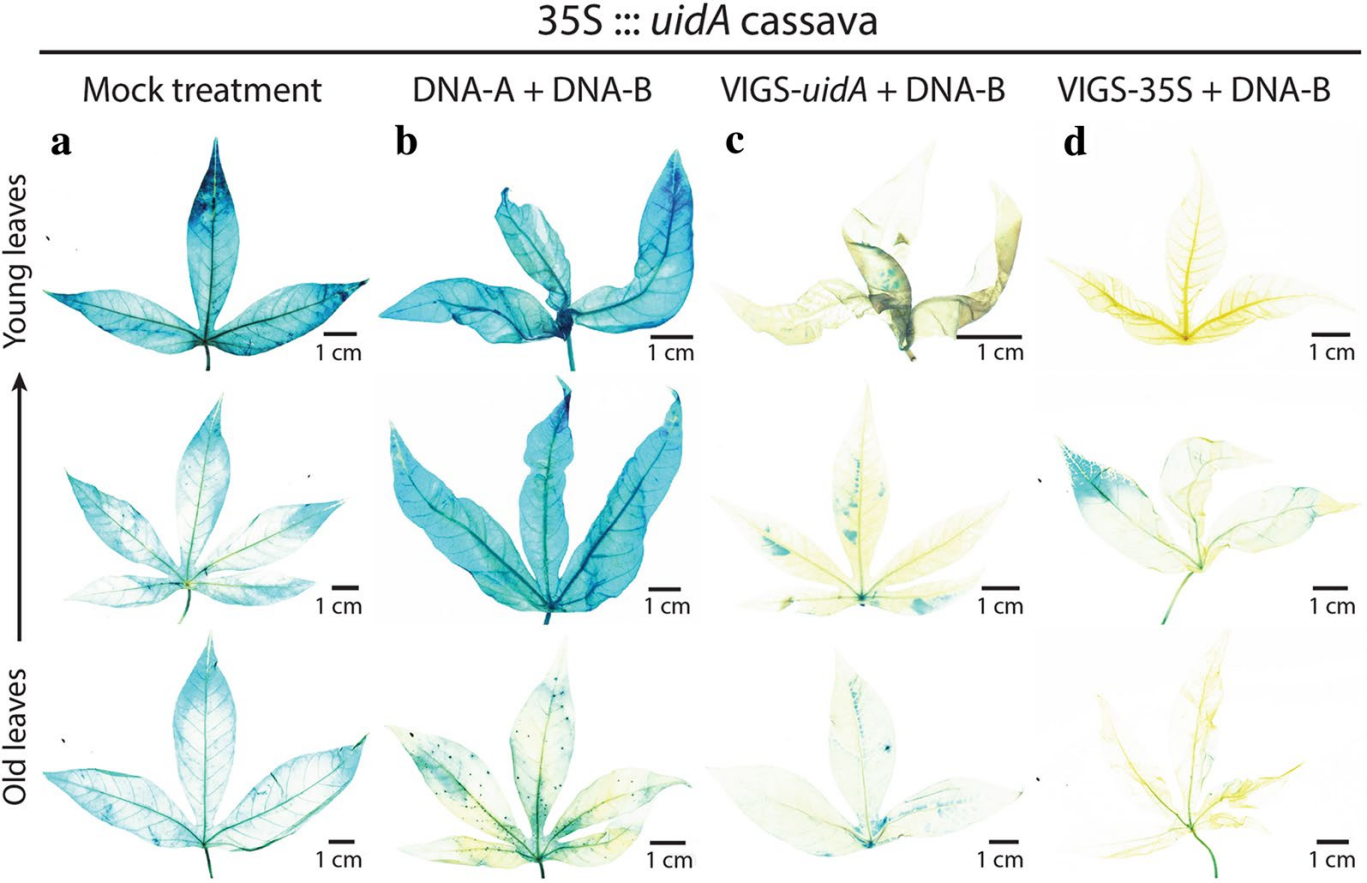
VIGS assays using leaves from infected transgenic 35S::*uidA* cassava plants. **a** Mock treatment, **b** infection with DNA-A + DNA-B, **c** infection with VIGS-*uidA* + DNA-B, **d** infection with VIGS-35S + DNA-B. GUS staining of representative leaves from VIGS-infected plants 2 months post-inoculation. One of the three biological replicates per treatment is shown. Infection rates are presented in Additional file 1: Table S1 A, E and F

In order to visualize gene silencing in both leaves and roots, we used a transgenic cassava line transformed with the pCambia1301 vector (GenBank: AF234297.1) containing a 35S::*uidA* gene construct. The 35S::*uidA* transgenic line expresses the *uidA* reporter gene encoding the β-glucuronidase enzyme (GUS) under the control of the CaMV 35S promoter. The 35S::*uidA* cassava plants were agro-inoculated with VIGS constructs carrying either a DNA fragment of the *uidA* coding sequence (VIGS-*uidA*) or DNA fragment of the 35S promoter (VIGS-35S). Both VIGS constructs resulted in high infection rates (Additional file 1: Table S1 E and F). We consistently observed higher GUS staining in young leaves as compared to old leaves in the control cassava plants (Fig. 3a and Additional file 1: Fig. S3A). Infection of 35S::*uidA* cassava leaves with the wild-type ACMV virus led to increased GUS staining of symptomatic leaves (Fig. 3b and Additional file 1: Fig. S3B). Visual observation of GUS silencing in leaves of plants infected with VIGS-*uidA* and VIGS-35S vectors suggested that the silencing of the *uidA* transgene was higher with the VIGS-35S vector (Fig. 3c, d and Additional file 1: Fig. S3C, D).

**Fig. 4:**
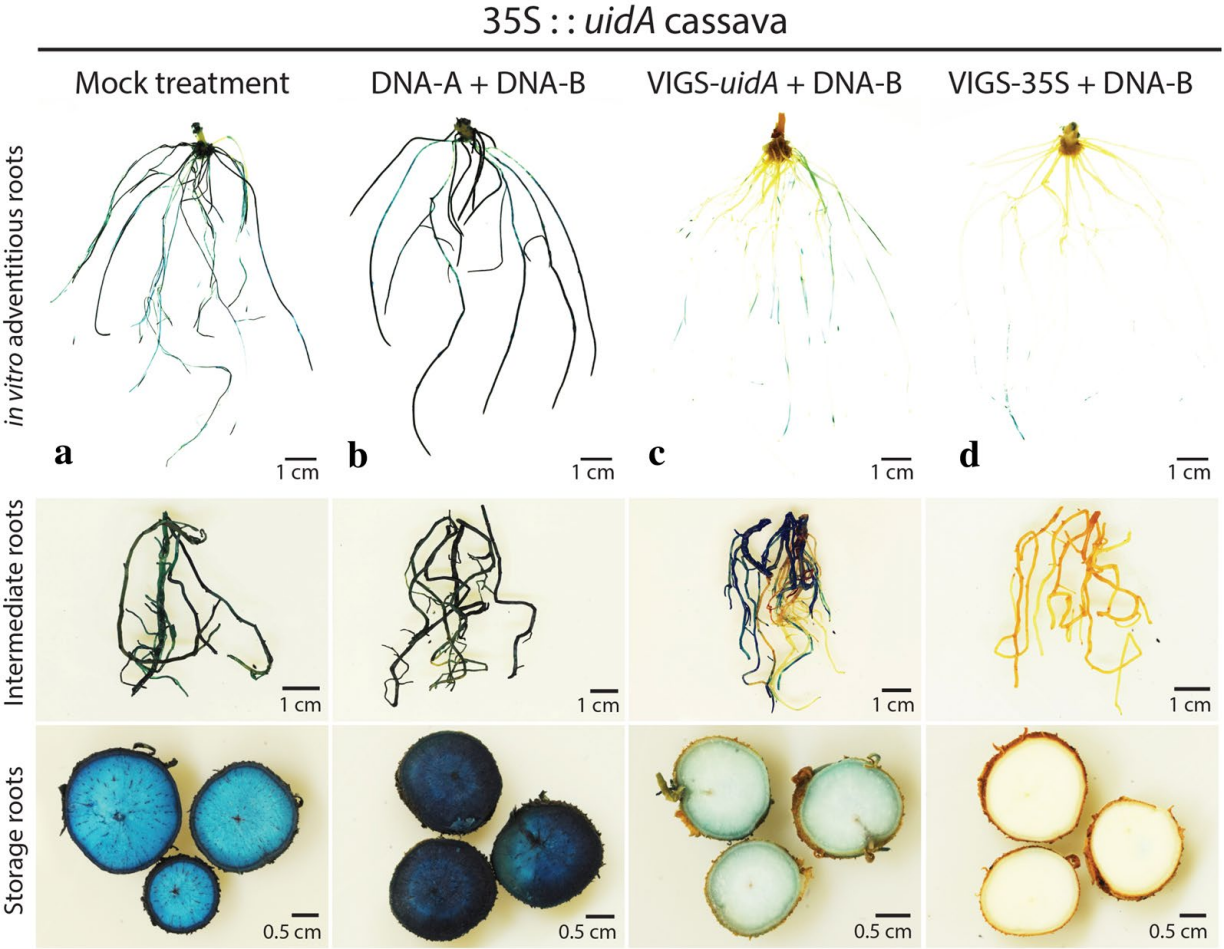
VIGS assays using roots of infected transgenic 35S::*uidA* cassava plants. **a** Mock treatment, **b** infection with DNA-A + DNA-B, **c** infection with VIGS-*uidA* + DNA-B, **d** infection with VIGS-35S + DNA-B. Adventitious roots from sterile stem cuttings of VIGS-infected cassava were grown in tissue culture and tested for GUS activity after 4 weeks. Intermediate and storage roots of VIGS-infected cassava plants grown in the greenhouse were taken from the soil and tested for GUS activity 6 months post-inoculation. One of the three biological replicates per treatment is shown. Infection rates are presented in Additional file 1: Table S1 A, E and F

We previously found that the 35S::*uidA* reporter gene is strongly expressed in fibrous roots of transgenic pCambia1301 cassava lines [12]. In order to assess the level of *uidA* silencing in the roots of 35S::*uidA* plants inoculated with the VIGS constructs, stem cuttings from the VIGS-*uidA* and VIGS-35S infected plants were propagated in vitro and adventitious roots emerging from these cuttings were tested for GUS activity. Infections with both VIGS-*uidA* and VIGS-35S resulted in *uidA* silencing in adventitious roots (Fig. 4 and Additional file 1: Fig. S4). The GUS-staining was consistently weaker in the roots of the VIGS-35S infected plants as compared to the roots of the VIGS-*uidA* infected plantlets (Fig. 4c, d and Additional file 1: Fig. S4C, D). The in vitro plantlets were subsequently transferred to the greenhouse and grown for 6 months to produce storage roots. Both VIGS-*uidA* and VIGS-35S vectors caused reduced GUS activity and staining in the fibrous and storage roots from VIGS-infected plants (Fig. 4 and Additional file 1: Fig. S5). As previously found for the in vitro adventitious roots (Fig. 4 and Additional file 1: Fig. S4), the VIGS-35S vector consistently caused stronger silencing of the *uidA* gene in both fibrous and storage roots as compared to VIGS-*uidA* vector.

### VIGS of endogenous genes in wild-type cassava

**Fig. 5:**
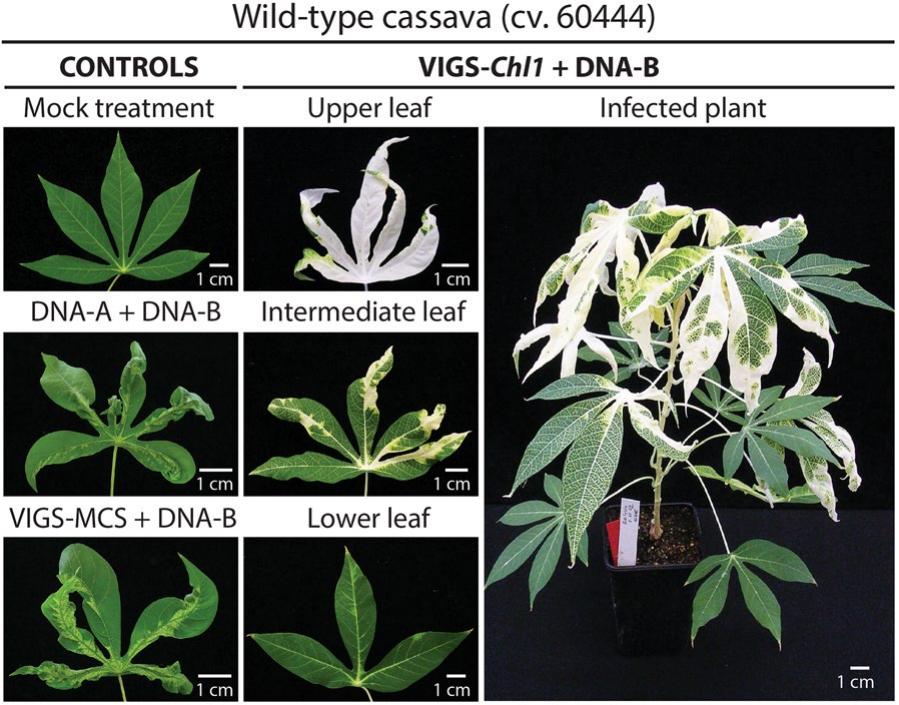
Summary of the VIGS assay for silencing of the *Chl1* gene in wild-type cassava. Representative images of control and infected leaves showing silencing of the *Chl1* gene encoding the Mg^2+^-chelatase enzyme (Manes.17G053100). Images were recorded 2 months post-inoculation. Infection rates are presented in Additional file 1: Table S1 D

**Fig. 6:**
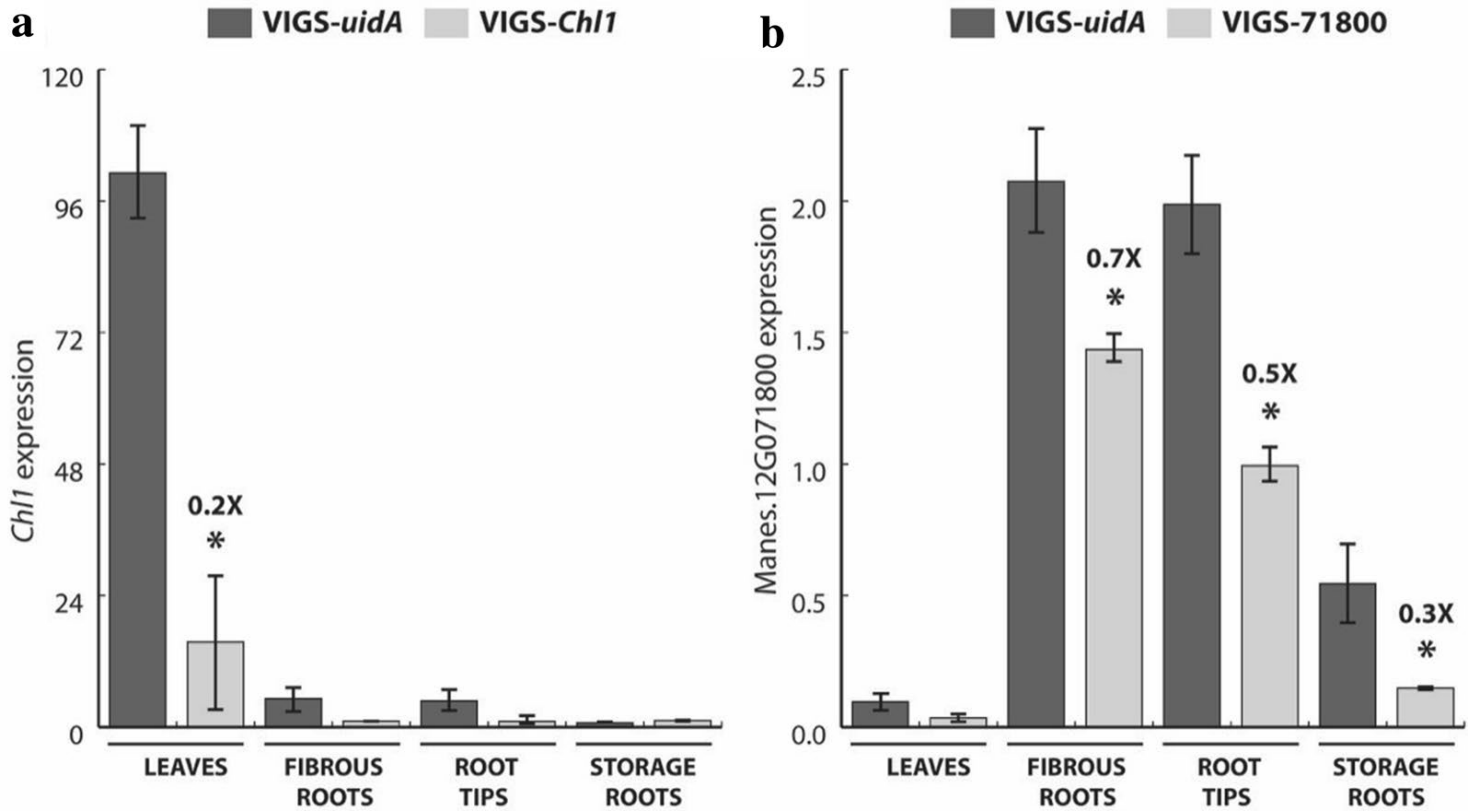
Evaluation of silencing in leaves, fibrous roots, root tips and storage roots for a leaf- and root-expressed gene in wild-type cassava. . Plants infected with VIGS constructs targeting different endogenous genes were evaluated by RT-qPCR 6 months p.i. **a** *Chl1* gene, coding for the Mg^2+^-chelatase (Manes.17G053100). **b** Lysin motif (LysM) receptor kinase gene (Manes.12G071800). Control plants were infected with the VIGS-*uidA* construct, targeting the *uidA* transgene. Bars represent means and standard deviations of expression levels calculated according to the Pfaffl method (2001) from three biological replicates displaying CMD symptoms (asterisks indicate significant differences in two-tailed *t* tests, *p *<0.05). The reference gene used for normalization is *PP2A* (Manes.09G039900). Fold change in gene expression levels as compared to the VIGS-*uidA* control samples is indicated above the bar for the biological samples displaying a significant reduction in gene expression. Infection rates are presented in Additional file 1: Table S1 D, E and G

Since the VIGS vectors could efficiently silence the *uidA* transgene in cassava plants, we also tested the VIGS vector to silence endogenous genes. We targeted the *Chl1* gene (Manes.17G053100), which encodes the first enzyme of the chlorophyll biosynthesis pathway (Mg^2+^-chelatase subunit I), because the pale phenotype in tissues that have lost the function of the enzyme can be easily observed [37]. Three to four weeks after agro-inoculation, both CMD symptoms and *Chl1* silencing were visible in infected cassava plants (Fig. 5 and Additional file 1: Fig. S6). The *Chl1* silencing pattern followed the viral movement through the stem, vascular tissue and leaves. The extent of silencing appears to be correlated with symptom severity. Silencing of the *Chl1* gene was maintained during 10 sequential multiplications of the original inoculated plants and was visible in plants propagated in vitro and in the greenhouse (Additional file 1: Fig. S7A). The *Chl1* silencing could also be transmitted from VIGS-inoculated 60,444 rootstocks to non-infected scions of the BRA222 cassava cultivar (Additional file 1: Fig. S7B). RT-qPCR analysis of VIGS-*Chl1*-infected 60,444 plants confirmed that *Chl1* expression was significantly down-regulated in intermediate leaves displaying clear CMD symptoms with patches of silenced and non silenced tissue areas (Fig. 6a). Thermotherapy of in vitro plantlets was effective in eliminating the VIGS-*Chl1* vector and restoring a fully green phenotype after a four-cycle treatment (Additional file 1: Fig. S7C). In addition, an endogenous root-expressed gene coding for a lysin motif (LysM) receptor kinase (Manes.12G071800) and present on a cassava genomic scaffold associated with the *CMD2* locus [38], was targeted with a VIGS construct containing a 500 bp fragment homologous to exons from the 5′ and 3′ ends of the Manes.12G071800 gene (Additional file 1: Table S1 G). A moderate and significant silencing (ranging from 1.4 to 3.7 fold as compared to control VIGS-*uidA* inoculated plants) of *Manes.12G071800* gene could be detected by RT-qPCR in fibrous roots, root tips and storage roots from symptomatic plants inoculated with the VIGS-71800 vector (Fig. 6b).

## Discussion

In the present work, we demonstrated that the expression of genes and transgenes can be effectively downregulated after infection with a modified ACMV agroclone in *Nicotiana benthamiana* and cassava leaves.

Using 35S::*uidA* cassava plants we observed that the *uidA* transgene can be silenced more effectively with a VIGS construct targeting the 35S promoter (VIGS-35S) than the coding sequence (VIGS-*uidA*). The efficacy of gene silencing by targeting a promoter has been previously reported for transgenic maize [39]. It remains to be demonstrated that the stronger silencing of the *uidA* gene in cassava plants inoculated with the VIGS-35S construct as compared to VIGS-*uidA* construct is caused by 35S promoter methylation and concomitant reduction of its transcriptional activity in transgenic cassava. The more intense GUS staining in leaves, fibrous roots and storage roots of 35S::*uidA* transgenic cassava infected with the non-modified virus also suggests a basal transgene silencing in this transgenic line, that is suppressed by the presence of ACMV-[NOg] and the production of viral suppressor(s) of RNA silencing, possibly AC2 and/or AC4 viral proteins [40].

Our results showed that an ACMV-based VIGS vector is also able to trigger silencing in cassava fibrous and storage roots. While visual observation of silencing in the transgenic 35S::*uidA* cassava line suggested a relatively strong silencing in cassava fibrous and storage roots, expression analysis of endogenous cassava genes revealed a moderate gene silencing in root tissues for the selected gene candidate.

Once infectious clones of *cassava brown streak virus*es (CBSVs) become available, they could also be developed into VIGS vectors to silence root-expressed genes in cassava, given their replication rate in cassava roots [41, 42]. However, development of CBSV-based VIGS vectors might require the use of attenuated CBSV isolates to avoid the interference of CBSV root symptoms in gene function analysis and phenotype evaluation. It should also be noted that geminivirus-based VIGS technique remains constrained to cassava genotypes susceptible to cassava geminiviruses. Therefore development of other VIGS vectors such as ipomovirus-based vectors could also be instrumental to expand the use of the VIGS technique to genotypes resistant or tolerant to geminiviruses.

## Conclusions

This VIGS protocol for cassava reported here allows cost-effective and high-throughput silencing of candidate genes by a simple agro-inoculation method in cassava. Given that most cassava genotypes are CMD susceptible, this reverse genetic tool can be widely used for gene validation studies. In addition, the VIGS observed in cassava fibrous and storage roots increases the versatility of this technique, which was originally developed and tested for leaf-expressed genes.

## Methods

### Genetic constructs

The cassava VIGS vector was designed by replacing 459 bp of the *AV1* sequence (without affecting the *AV2* gene) in the ACMV-[NOg] DNA A genome with a 30 bp multiple cloning site (Additional file 1: Fig. S1). The primers and plasmids used for the generation of the VIGS construct are listed in the Additional file 1: Tables S1 and S2. Sequence from the *Chl1* first exon (Manes.17G053100, 454 bp) was kindly provided by Stephan Winter (DSMZ, Germany). The cassava genome assembly employed to design the DNA fragments for specific gene silencing was based on the cassava AM560 reference genome version 4.1 [17]. DNA fragments with flanking *Spe* I and *Kpn* I restriction sites were first ligated in pJET1.2 (Life Technologies) and subsequently sequenced. After sequence confirmation, the fragments were mobilized into the VIGS vector digested with the *Spe* I and *Kpn* I restriction enzymes. The final constructs were transferred to *Agrobacterium tumefaciens* AGL1 competent cells using electroporation.

### Plant material and transgenic lines

All the experiments were performed using the cassava cultivar 60,444, except for the grafting experiment in which the BRA222 genotype was used. The 35S:::*uidA* cassava transgenic plant was generated using the pCambia 1301 vector (GenBank: AF234297.1) and the genetic transformation protocol previously established in our laboratory [9, 12]. The transfer of greenhouse material to in vitro conditions and thermotherapy treatment of in vitro material for virus elimination was performed as described in the CIAT handbook (Mafla et al. [43]. Briefly, in vitro cuttings were incubated at 37 °C for 12 days and emerging shoots were employed as new cuttings. The procedure was repeated three times.

### VIGS assay in cassava and *N. benthamiana*

For VIGS inoculation experiments, 6 weeks-old cassava plants grown from stem cuttings and 3 weeks-old *N. benthamiana* plants grown from seeds in the greenhouse were used for agroinoculation.

*Agrobacterium tumefaciens* LBA4404 and AGL1 strains containing the different VIGS constructs were refreshed from glycerol stocks on YEBA plates (5 g/L beef extract, 1 g/L yeast extract, 5 g/L peptone, 5 g/L sucrose, 0.5 g/L MgCl_2_, 1.5% agar) containing 100 mg/L carbenicillin (C), 20 mg/L rifampicin (R) and 50 mg/L kanamycin (K), and grown for 48 h at 28 °C in the dark. Fifty-ml sterile tubes containing 5 mL of YEB + C100/R20/K50 were inoculated with the different clones separately and grown for 24–36 h at 28 °C and 150 rpm until they reached an OD_600 nm_ > 1. Two mL of the starter cultures were added to 1 L Erlenmeyer flasks containing 100 mL of YEB + C100/R20/K50. Cultures were grown for 24–36 h at 28 °C and 150 rpm until they reached an OD_600 nm_ = 1.5–2. Agrobacteria suspensions were centrifuged in 250 mL bottles, previously sterilized with 70% EtOH and rinsed with sterile deionized water. After centrifugation at 4000*g* for 20 min at room temperature, the bacterial pellets were carefully re-suspended in 300 mL of sterile deionized water at room temperature, and centrifuged again as indicated previously. The washed bacterial pellets were re-suspended carefully in 10–20 mL of LB and OD_600 nm_ adjusted with LB to a final value of 4. A defined volume of suspension of Agrobacterium carrying the DNA-A vector (or VIGS vector) combined with the same volume of suspension of Agrobacterium carrying the DNA-B vector was prepared. After addition of acetosyringone to a final concentration of 200 μM, the bacterial suspensions were kept at room temperature for 3 h at 50 rpm. To agro-inoculate the plants, we adapted a protocol previously established in the laboratory to screen for ACMV resistance [24]. A 1 mL insulin syringe (0.33 mm/29 G/12.7 mm) was used to inject agrobacteria suspension at least three times near the axillary buds without damaging the meristems and the same syringe is used to gently puncture the stem 5–7 times. Inoculated plantlets were kept overnight at 20–24 °C in the dark and then placed in the greenhouse (28 °C, 16-h day length, 22 klx, 50% humidity). Alternatively, when more than 10 plants for a particular construct were agro-inoculated, we employed a high-throughput option of this protocol: the upper leaves were removed and a 21G needle, previously soaked in agrobacteria suspension, was used to puncture the stem at least 3 times prior to dipping the 4–5 week-old plantlets into a 50 mL tube containing the agrobacteria suspension for 30 s. Plantlets were subsequently allowed to recover for 4 days in a high humidity chamber (28 °C, 16-h day length, 22 klx, >80% humidity) before being placed under greenhouse conditions (28 °C, 16-h day length, 22 klx, 50% humidity). All experiments included at least three biological replicates per treatment.

### GUS staining and image processing

Plant tissue samples were first incubated in pre-cooled (− 20 °C) 90% acetone for 2 h at − 20 °C. The material was rinsed with freshly prepared GUS buffer (100 mM NaH_2_PO_4_ pH 7, 10 mM EDTA-Na_2_, 0.5 mM K_4_[Fe(CN)_6_], 0.5 mM K_3_[Fe(CN)_6_], 0.1% Triton-X100) [44], and then incubated for 20 min in GUS buffer at room temperature. The buffer was discarded and fresh GUS buffer containing 1 mM X-Gluc was added. The staining was performed for 24 h at 37 °C in the dark. Every step in the staining protocol included 20 min of vacuum infiltration at room temperature. For the staining of fibrous roots from in vitro plants, the agar media was carefully removed with tap water. For staining intermediate and storage roots from greenhouse plants, the X-Gluc concentration was increased to 2 mM. After the 24 h incubation, the staining solution was discarded and the samples were de-stained three times and stored in 70% EtOH. After 5 min of rehydration in deionized water, leaves and fibrous roots from in vitro plantlets were photographed on a light chamber with a Nikon D700 camera (VR AF-S Micro Nikkor 105 mm 1:2:8 G ED objective), and image brightness was adjusted using Adobe Photoshop software. Intermediate and storage roots were photographed with the same equipment under standard room illumination conditions and images were not adjusted for brightness.

### RNA extraction and RT-qPCR

Three symptomatic intermediate leaves were pooled for each biological replicate and total RNA was extracted according to the protocol by Soni and Murray [45]. Fibrous and storage roots from plants displaying leaf symptoms were collected for RNA extraction. Total RNA from fibrous roots was prepared using the Invitrap Spin Plant RNA Mini Kit (Stratec Molecular GmbH, Berlin, Germany) according to the manufacturer’s instructions and using the “RP lysis” solution option. Total RNA from storage roots was extracted using a protocol established for nucleic acid extraction from pineapple samples [46]. All RNA samples were quality checked using the NanoDrop system (Thermo Scientific, Wilmington, USA). RNA samples were treated with DNase I and converted to cDNA using the RevertAid First Strand cDNA Synthesis Kit (Thermo Scientific, Wilmington, USA) according to the manufacturer’s instructions. qPCR was performed using the 7500 Fast Real-Time PCR System (Applied Biosystems) and 5–50 ng of cDNA, 1 μM of primers and Fast SYBR Green Master Mix (Applied Biosystems) in a final volume of 20 μL. Each cDNA sample was checked for DNA contamination by including in parallel a total RNA sample treated with DNAse I as blank for qPCR. The RT-qPCR analysis included at least two technical replicates per sample. Relative gene expression was calculated using the [47] method and the *PP2A* gene (Manes.09G039900) expression values for normalization [42].

### Statistical analysis

Differences in gene expression levels measured by RT-qPCR, were analyzed using two-tailed *T* tests.

## Additional file

**Additional file 1**. Supplementary figures and tables.

## Abbreviations

ACMV: African Cassava Mosaic Virus
C100: 100 mg/L carbenicillin
CaMV: cauliflower mosaic virus
CBSVs: cassava brown streak viruses
CMD: cassava mosaic disease
CR: common region
K50: 50 mg/L kanamycin
LB: left border
MCS: multiple cloning site
p.i.: post infection
R20: 20 mg/L rifampicin
RB: right border
VIGS: virus-induced gene silencing
