# Supplementary material for: Cassava geminivirus agroclones for virus-induced gene silencing in cassava leaves and roots
Source: Plant Methods. 2018 Aug 27;14:73. doi: 10.1186/s13007-018-0340-5 (PMC6109987; doi:10.1186/s13007-018-0340-5)
Supplement: Supplementary file 1 — Additional file 1. Supplementary figures and tables. [file 13007_2018_340_MOESM1_ESM.pdf]

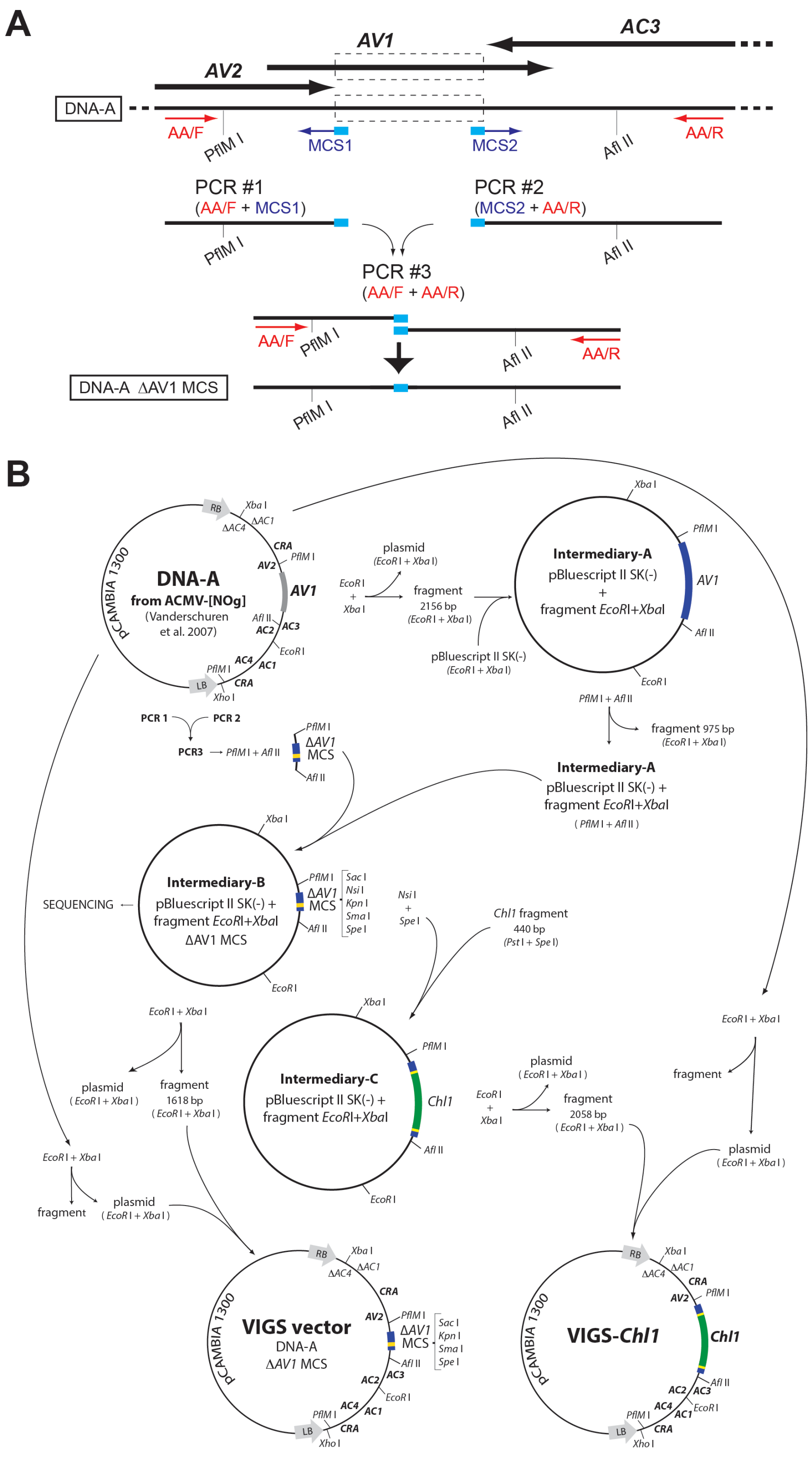

Fig. S1 Construction of the VIGS vector.

A) PCR strategy for removal of a fragment of the *AV1* gene in ACMV-[NOg] and insertion of a multiple cloning site (MCS). *CRA*: Common Region of DNA-A; *AV1*: coat protein gene; *AV2*: Protein V2 gene; *AC3*: Replication enhancer protein gene; *AC2*: Transcriptional activator protein gene; *AC1*: Replication associated protein gene; *AC4*: RNA silencing suppressor gene.

B) Cloning steps to generate the VIGS vector and the VIGS-*Chl1* construct targeting the gene encoding the cassava Mg^{2+}-chelatase enzyme (Manes.17G053100).

A

```
Identities = 144/170 (84%)

cassava              57 cctgaagaaggggagctcaggccacagctacttgatagatttggaatgcatgcacaagta 116
                        ||||||||||| || || |||||||| || |||||| |||||||||||||||| |||||
N. benthamiana     4160 cctgaagaaggagaacttaggccacaacttcttgatcgatttggaatgcatgcccaagtg 4219

cassava             117 ggaactgttaaagatgcggagctgagagtgaagatagttgaagaaagaggccgttttgat 176
                        || || || | |||||| ||||||||||| ||||| ||||| |||||||  |||||||||
N. benthamiana     4220 gggaccgtgagagatgcagagctgagagtaaagatcgttgaggaaagagctcgttttgat 4279

cassava             177 aaaaacccaaaagaatttcgtgattcttacaaggcagagcaagagaagct 226
                        || ||||| || ||||| || || || ||||||||||||||||| |||||
N. benthamiana     4280 aagaaccccaaggaattccgggagtcatacaaggcagagcaagaaaagct 4329
```

B

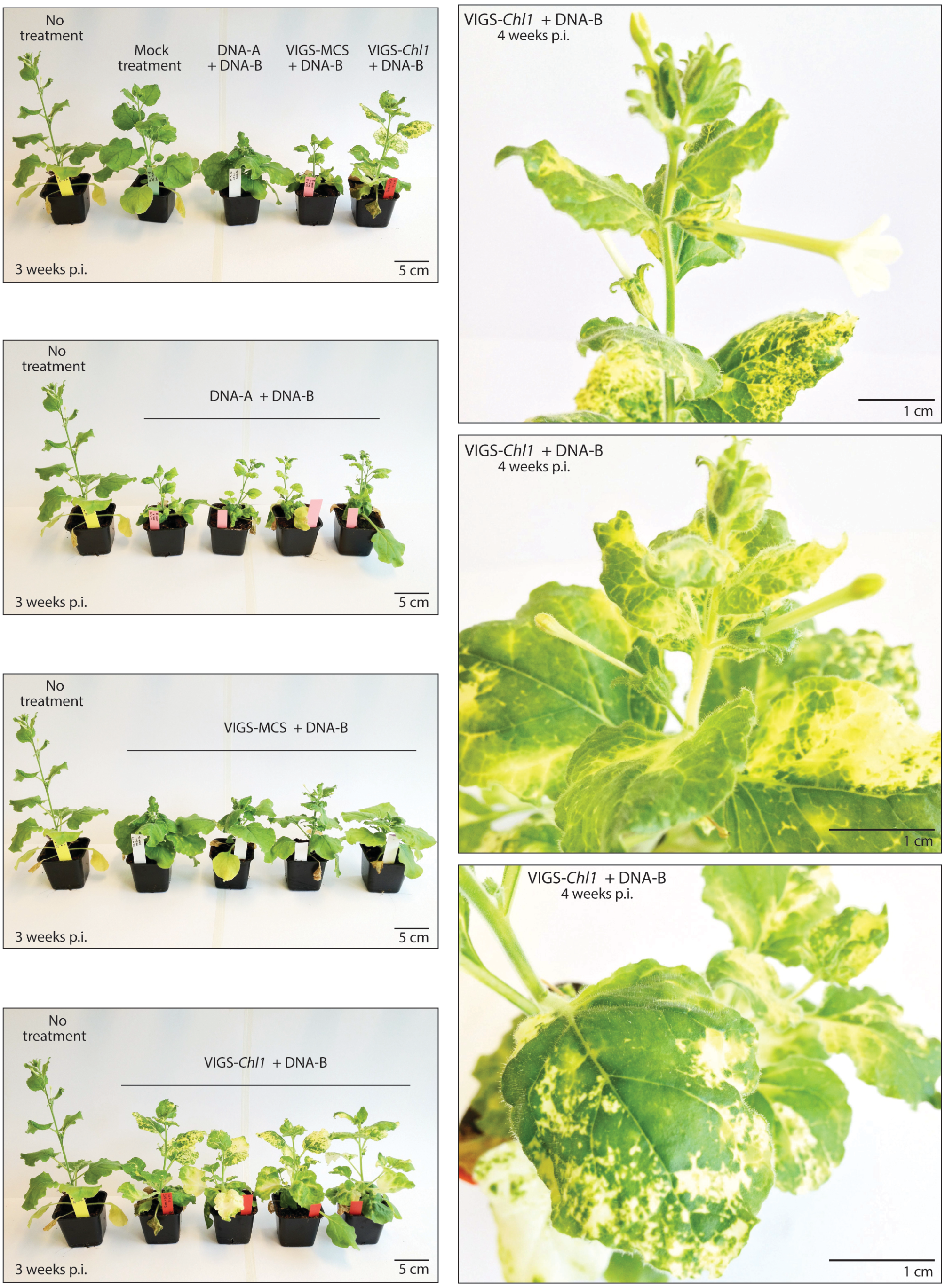

Fig. S2 Validation of the VIGS vector functionality in *N. benthamiana*.
A) Alignment of the cassava *Chl1* gene (Manes.17G053100) fragment included in the VIGS-*Chl1* construct with the *N. benthamiana Chl1* homologue.
http://solgenomics.net/organism/Nicotiana_benthamiana/genome
B) Examples of the VIGS phenotype in *N. benthamiana* targeting the *Chl1* gene.

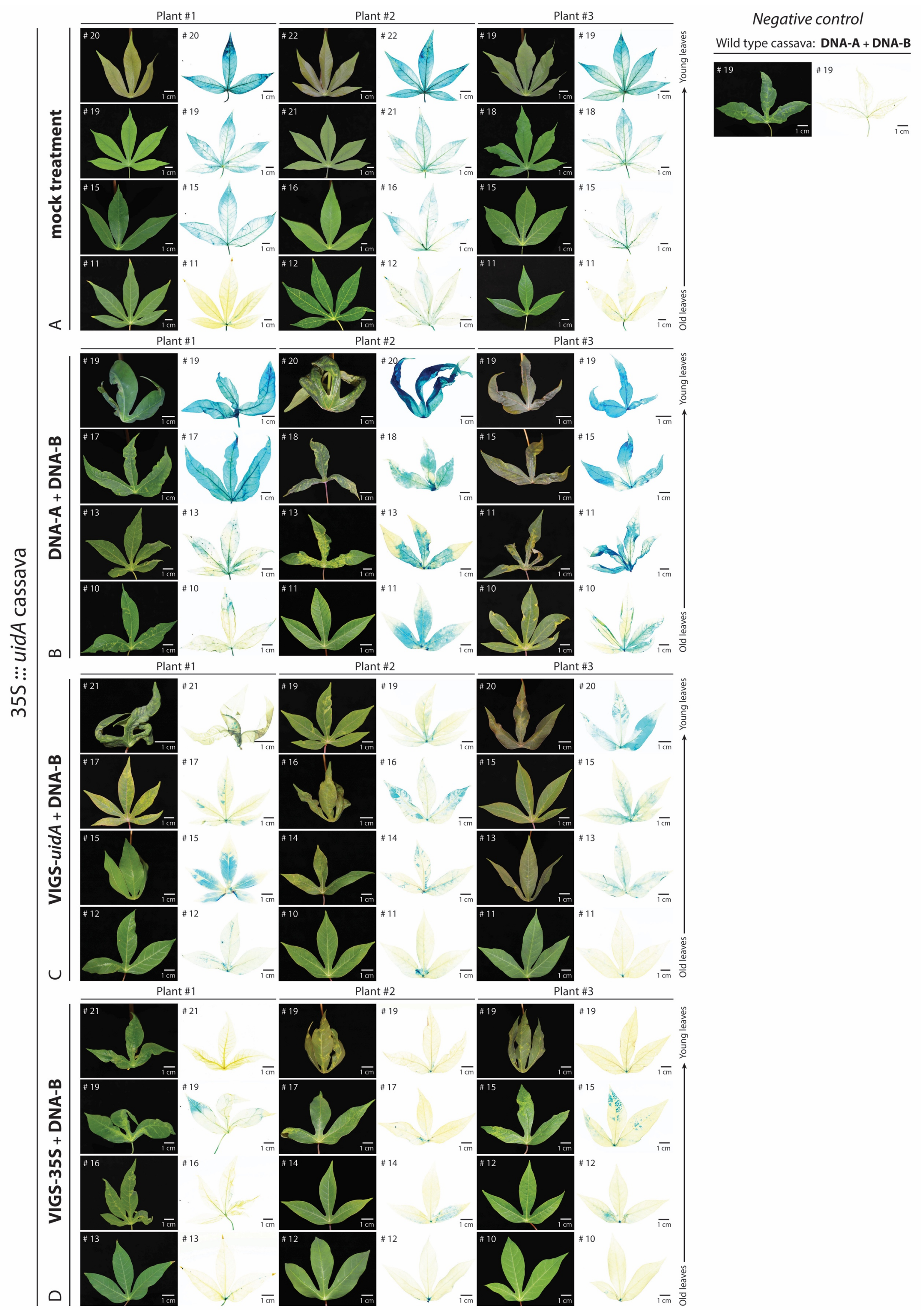

Fig. S3 VIGS assays targeting the *uidA* gene in transgenic 35S::*uidA* cassava leaves. GUS staining of leaves from VIGS-infected plants two months post-inoculation. The leaf number and images before GUS staining are shown for each sample and leaf phenotypes of three biological replicates for each treatment.

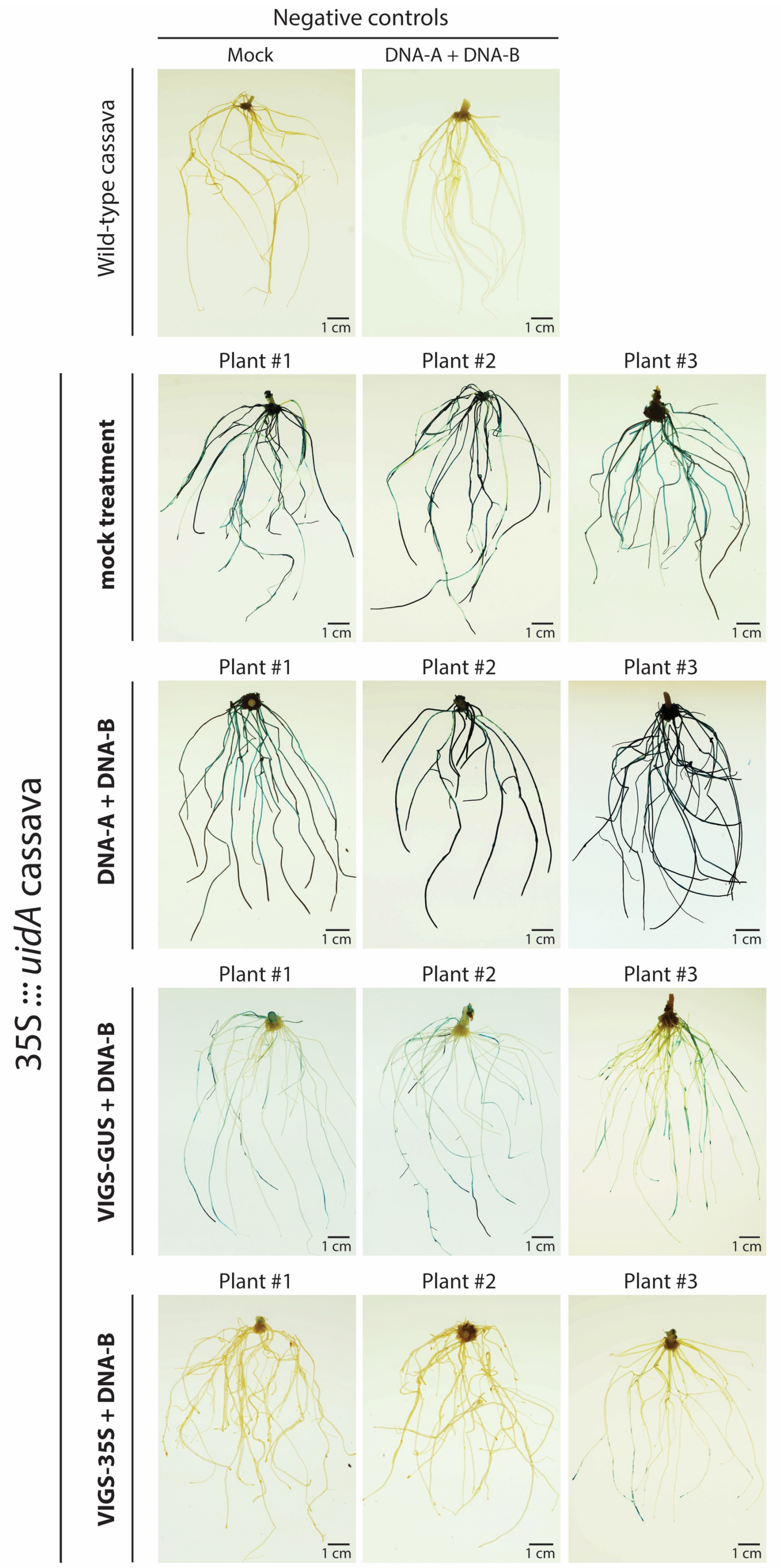

Fig. S4 VIGS assays targeting the *uidA* gene in transgenic 35S::*uidA* cassava roots. Sterile stem cuttings from VIGS-infected cassava plants were grown in tissue culture and new roots tested for GUS activity after 4 weeks. Phenotypes of three biological replicates for each treatment are shown.

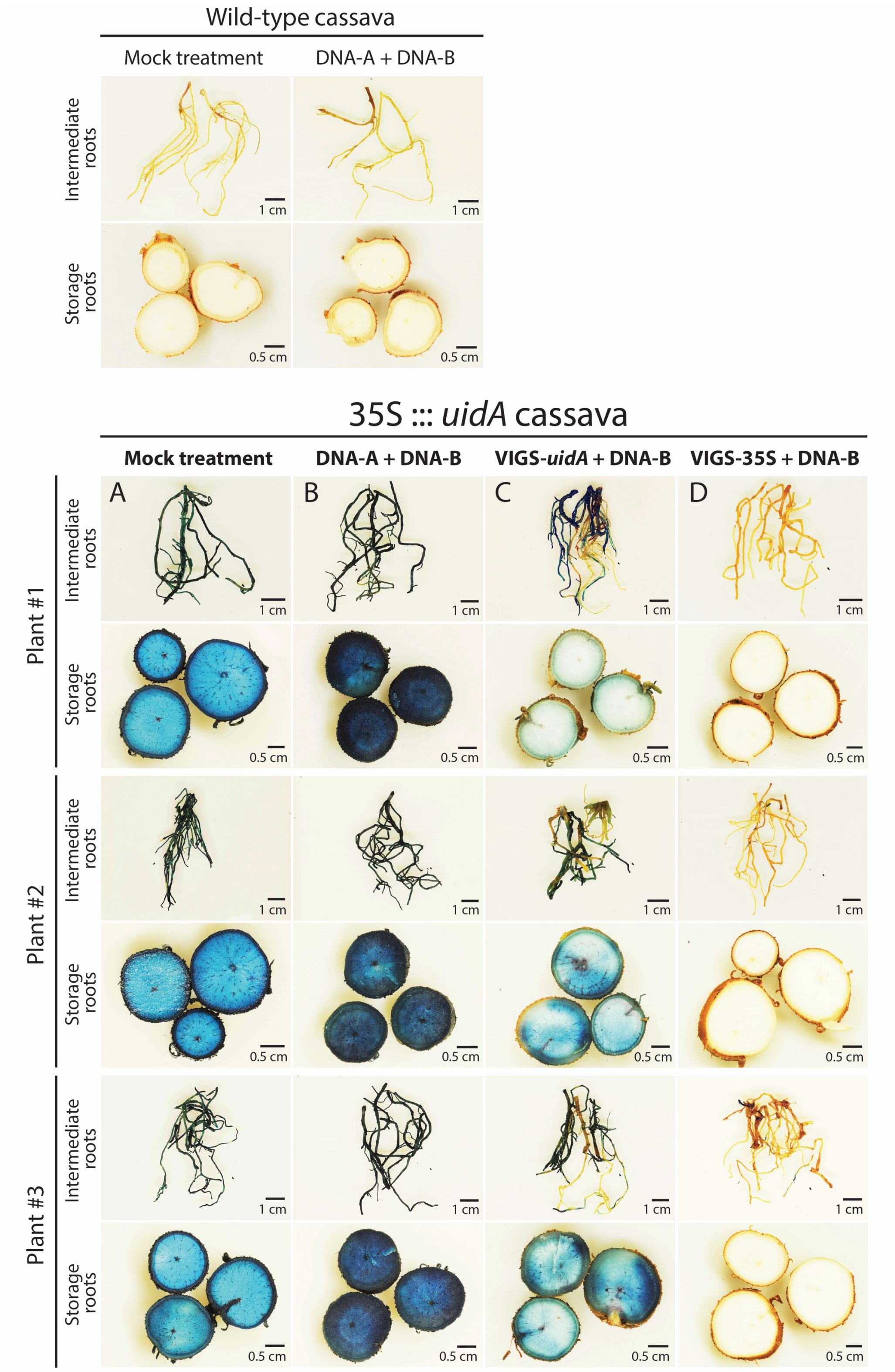

Fig. S5 Silencing in transgenic 35S::*uidA* cassava intermediate and storage roots. VIGS-infected cassava plants in the greenhouse were analyzed for GUS activity in roots six months post-inoculation. Phenotypes of three biological replicates for each treatment are shown.

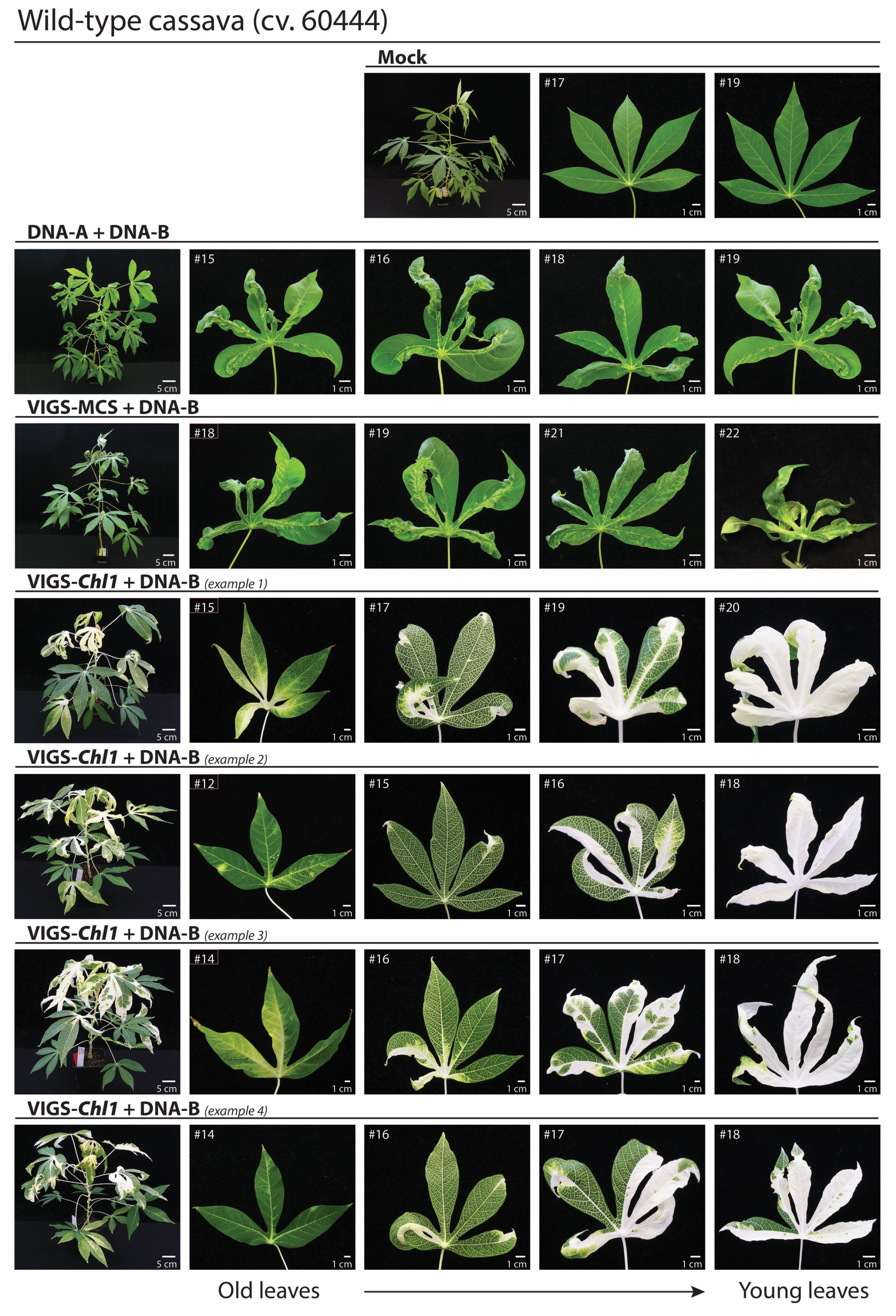

Fig. S6 VIGS assay targeting the *ChlI* gene in wild-type cassava. Examples of plants and leaves showing the silencing of the *ChlI* gene encoding the Mg^{2+}-chelatase enzyme (Manes.17G053100). Images were recorded two months post-inoculation. The leaf number is indicated in each case.

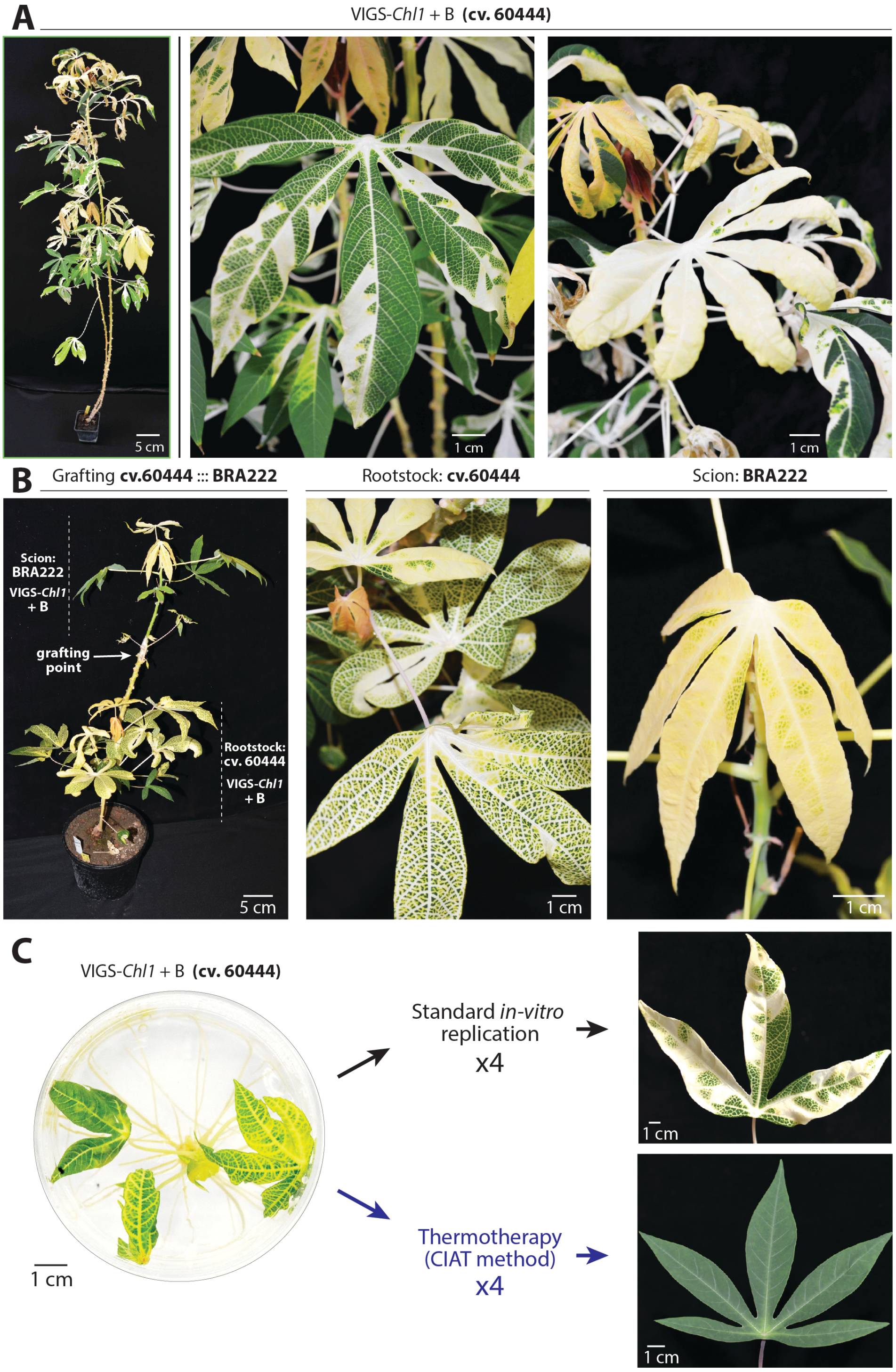

Fig. S7 Durability of the *Chl1*-silencing phenotype after grafting and removal of the VIGS vector by thermotherapy.

A) Phenotype of a plant infected with the VIGS-*Chl1* + B construct, one year post-inoculation.

B) Transfer of VIGS-*Chl1* + B from infected rootstock (cultivar 60444) to scion (cultivar BRA222) by grafting.

C) Removal of the VIGS-*Chl1* + B construct by thermotherapy, according to the CIAT method (Mafla et al. 2010). After 4 cycles of thermotherapy (or control *in-vitro* replications), plants were transferred to the greenhouse and photographed.

Table S1. Summary of genetic constructs and infection rates of VIGS vectors. *n* represents the number of inoculated plants.

	**Name** (size)	**Backbone** (size)	**Insert** (size)	**Purpose**	**Infection rate** by co-inoculation with DNA-B (*Agrobacterium* strain used)
**A**	**DNA-A** (10,297 bp)	pCambia 1300 (6,407 bp)	DNA-A tandem repeats from the ACMV-NOg isolate (Vanderschuren et al. 2009) (3,890 bp)	Construction of the VIGS vector and used as control in VIGS assays.	Cassava (LBA4404)<br>Assay #1 (n=3): 100%<br>Assay #2 (n=5): 100%<br>Cassava (AGL1)<br>Assay #1 (n=5): 100%<br>Assay #2 (n=3): 100%<br>Assay #3 (n=3): 100%<br>Assay #4 (n=4): 100%<br>Assay #5 (n=3): 100%<br>Assay #6 (n=5): 100%<br>*N. benthamiana* (LBA4404)<br>Assay #1 (n=4): 100%<br>Assay #2 (n=4): 100%
**B**	**DNA-B** (10,283 bp)	pCambia 1300 (6,407 bp)	DNA-B from the ACMV-NOg isolate (Vanderschuren et al. 2009) (3,876 bp)	This plasmid was co-inoculated with the DNA-A or VIGS constructs.	-
**C**	**VIGS-MCS** (9,766 bp)	Modified DNA-A: VIGS vector including a Multiple Cloning Site (MCS). (9,766 bp)	Empty VIGS vector. (no insert)	This plasmid was used as recipient plasmid for the below-mentioned gene fragments.	Cassava (LBA4404)<br>Assay #1 (n=5): 0%<br>Assay #2 (n=8): 0%<br>Cassava (AGL1)<br>Assay #1 (n=3): 33%<br>Assay #2 (n=11): 27%<br>*N. benthamiana* (LBA4404)<br>Assay #1 (n=4): 100%<br>Assay #2 (n=4): 100%
**D**	**VIGS-*ChlI*** (10,214 bp)	VIGS-MCS (9,766 bp)	Middle part of the *ChlI* first exon (Manes.17G053100) (454 bp)	Silencing of the Mg^{2+}-chelatase gene and inhibition of chlorophyll synthesis. Example of VIGS for endogenous genes.	Cassava (LBA4404)<br>Assay #1 (n=4): 0%<br>Assay #2 (n=16): 0%<br>Cassava (AGL1)<br>Assay #1 (n=13): 92%<br>Assay #2 (n=6): 100%<br>*N. benthamiana* (LBA4404)<br>Assay #1 (n=3): 100%<br>Assay #2 (n=4): 100%<br>Assay #3 (n=4): 100%
**E**	**VIGS-*uidA*** (10,220 bp)	VIGS-MCS (9,766 bp)	5' end of the *uidA* gene (460 bp)	VIGS assays targeting the coding sequence of the transgene 35S::*uidA* in transgenic cassava plants, and control construct for VIGS assays in wild-type plants.	Cassava (AGL1)<br>Assay #1 (n=9): 89%<br>Assay #2 (n=4): 75%
**F**	**VIGS-35S** (10,242 bp)	VIGS-MCS (9,766 bp)	-470 > +1 region of the 35S promoter (482 bp)	VIGS assays targeting the promoter of the transgene in 35S::*uidA* in transgenic cassava plants.	Cassava (AGL1)<br>Assay #1 (n=6): 100%<br>Assay #2 (n=8): 100%
**G**	**VIGS-*71800*** (10,260 bp)	VIGS-MCS (9,766 bp)	500 bp fragment of the *Manes.12G071800.1* targeting exon 1, 10, 11 and 12 (5'end and 3'end of the CDS)	Example of VIGS for endogenous genes.	Cassava (AGL1)<br>Assay #1 (n= 8): 100%

Table S2. Summary of DNA primers.

Name	Sequence (5' – 3')	Purpose
AA/F	ACTGGTGAATGAGTTTCCAGACTCGGT	Construction of the VIGS vector
AA/R	TCCAACACGAAATACGGGAAACCC	Construction of the VIGS vector
MCS1	ACTAGTCCCGGGGGTACCATGCATGAGCTCA GGTATGTCTGGGCTTCTATACATCCTGTACAT	Construction of the VIGS vector
MSC2	GAGCTCATGCATGGTACCCCCGGGACTAGTG ACAGTATTGGCAATTAATAAACATTGAATTTT ATTTCA	Construction of the VIGS vector
ACMV-fw	CAA TTT CCA CCC CAA CAT TCA	ACMV (and VIGS constructs) detection
ACMV-rv	GCG TAA GCA TCA TTC GCT GAT	ACMV and (VIGS constructs) detection
Chl1-cDNA fw	TTTTGGGTTCTCGGCGTTTCT	Quantification of *Chl1* (*Manes.17G053100*) expression by qPCR.
Chl1-cDNA rv	CATGAAATTGGGACCTCCCTT	Quantification of *Chl1* (*Manes.17G053100*) expression by qPCR.
21633 fw	TTGACGACGGTTTCGTGG	Quantification of *Manes.12G071800* expression by qPCR.
21633 rv	CCGGAGCGGGTACGTAAT	Quantification of *Manes.12G071800* expression by qPCR.
PP2A-cDNA fw	TGCAAGGCTCACACTTTCATC	Quantification of *PP2A* (*Manes.09G039900*) expression by qPCR.
PP2A-cDNA rv	CTGAGCGTAAAGCAGGGAAG	Quantification of *PP2A* (*Manes.09G039900*) expression by qPCR.
